# Protective Effects of Ethyl Pyruvate and Rutin Hydrate Against Colistin-Induced Nephrotoxicity in Rats

**DOI:** 10.3390/ph19091417

**Published:** 2026-09-08

**Authors:** Mehmet Nuri Gorduk, Ilker Kelle, Meral Erdinc, Meryem Seyda Kaya, Firat Asir

**Affiliations:** 1 Department of Medical Pharmacology, Faculty of Medicine, Dicle University, Diyarbakir 21280, Türkiye; 2 Department of Public Health, Faculty of Medicine, Mardin Artuklu University, Mardin 47200, Türkiye; 3 Department of Pharmacology, Faculty of Pharmacy, Dicle University, Diyarbakir 21280, Türkiye; meryemseydakaya@gmail.com; 4 Department of Histology and Embryology, Faculty of Medicine, Dicle University, Diyarbakir 21280, Türkiye; firatasir@gmail.com

**Keywords:** colistin, ethyl pyruvate, nephrotoxicity, rutin hydrate, Wistar albino rats

## Abstract

**Background**: Colistin is increasingly being used as a last-line treatment option for multidrug-resistant Gram-negative infections; however, its clinical use remains limited due to its nephrotoxic side effects. This study investigated the potential nephroprotective effects of ethyl pyruvate (EP) and rutin hydrate (RH), alone and in combination, against colistin-induced renal damage. **Methods**: Forty-nine adult male Wistar albino rats were allocated into seven groups, and all agents were administered intraperitoneally once daily for seven days. On the eighth day, one kidney of each animal was perfused using a Langendorff system to measure perfusion pressure, while samples from the contralateral kidney and blood were used for biochemical, molecular, and histopathological analyses. **Results**: In the colistin group, urea, creatinine, total oxidant status, and perfusion pressure were significantly higher than in the control, EP, and RH groups (*p* < 0.05). Oxidative stress index, tissue MDA, KIM-1, caspase-3, and PUMA levels were also markedly increased. All of these parameters decreased in the treatment groups, with the lowest values for most parameters in the group receiving both EP and RH, which also showed the best-preserved renal architecture. **Conclusions**: These findings suggest that EP and RH may be promising adjuvant agents against colistin-induced nephrotoxicity. Combined EP and RH co-treatment showed numerically greater protection across most assessed parameters, although an overall advantage over single-agent co-treatment was not statistically established.

## 1. Introduction

The discovery of antibiotics in the first half of the 20th century marked groundbreaking advancements in human health and represented a pivotal turning point in the fight against infectious diseases. This development has led to a remarkable reduction in infection-related morbidity and mortality while also contributing significantly to the increase in average life expectancy [1]. However, the widespread and often inappropriate use of antibiotics has led to the emergence of bacterial resistance, thereby diminishing the effectiveness of existing treatments. Alexander Fleming, in his 1945 Nobel Lecture, had already cautioned against this potential risk, emphasizing the dangers of misuse and the possible consequences for future generations [2]. In recent years, the increasing resistance rates among Gram-negative pathogens have severely limited the number of available treatment options. Consequently, several antibiotics that have been abandoned in the past due to serious adverse effects have been reintroduced into clinical practice. One such antibiotic is colistin, which was largely discontinued in the 1970s because of its notable side effects, particularly nephrotoxicity. Today, however, it has regained prominence as a last-resort therapeutic agent against multidrug-resistant and extensively drug-resistant pathogens. In particular, the use of colistin has increased markedly in recent decades because of its effectiveness against resistant Gram-negative bacteria such as *Acinetobacter* spp., *Pseudomonas aeruginosa*, and *Klebsiella* spp. [3,4,5]. Parallel to the increasing frequency of colistin use, its major adverse effects—most notably nephrotoxicity—which limit its clinical application, have once again become significant clinical concerns. In an effort to prevent this serious adverse outcome, numerous preclinical and experimental studies have been conducted. In particular, various pharmacological agents, antioxidant molecules, herbal extracts, and biological compounds have been investigated for their potential to mitigate colistin-induced nephrotoxicity, and research in this field is still ongoing [6].

Nephrotoxicity, one of the most serious adverse effects of colistin, has been clearly demonstrated in both clinical and experimental studies. Meta-analyses have reported that colistin monotherapy carries a greater risk of nephrotoxicity than combination therapies do [7,8]. Furthermore, kidney injury associated with colistin therapy typically develops within the first week of treatment and is reversible in most cases [7,9,10].

Ethyl pyruvate, a derivative of pyruvate—the end product of glycolysis—is more stable than pyruvate in aqueous solution and has demonstrated tissue-protective effects in various experimental models. It has been reported to provide protective effects in numerous conditions, including hemorrhagic shock, sepsis, and septic shock; ischemia–reperfusion injuries; burn and radiation injuries; acute pancreatitis; hepatic and renal injuries; viral myocarditis; and traumatic brain injuries, mainly through its antioxidant, anti-inflammatory, and antiapoptotic properties [11,12].

On the other hand, rutin hydrate, a naturally occurring flavonoid, is widely found in many fruits, vegetables, leaves, and seeds, particularly in apples, onions, buckwheat, blackberries, and tea. It has demonstrated tissue-protective potential in various disease models, with reported antioxidant, anti-inflammatory, antimicrobial, anticancer, neuroprotective, nephroprotective, and hepatoprotective effects across different tissues and organ systems [13,14,15].

Despite the reported protective potential of ethyl pyruvate and rutin hydrate in various experimental models, their effects on colistin-induced nephrotoxicity have not yet been investigated. This study aimed to investigate the potential protective effects of ethyl pyruvate and rutin hydrate against colistin-induced acute kidney injury in Wistar albino rats. In this context, treatment effects were comprehensively evaluated through histopathological assessments, biochemical analyses, and examination of oxidative stress and apoptosis markers.

## 2. Results

### 2.1. Serum Biochemical and Oxidative Stress Findings

**Figure 1:**
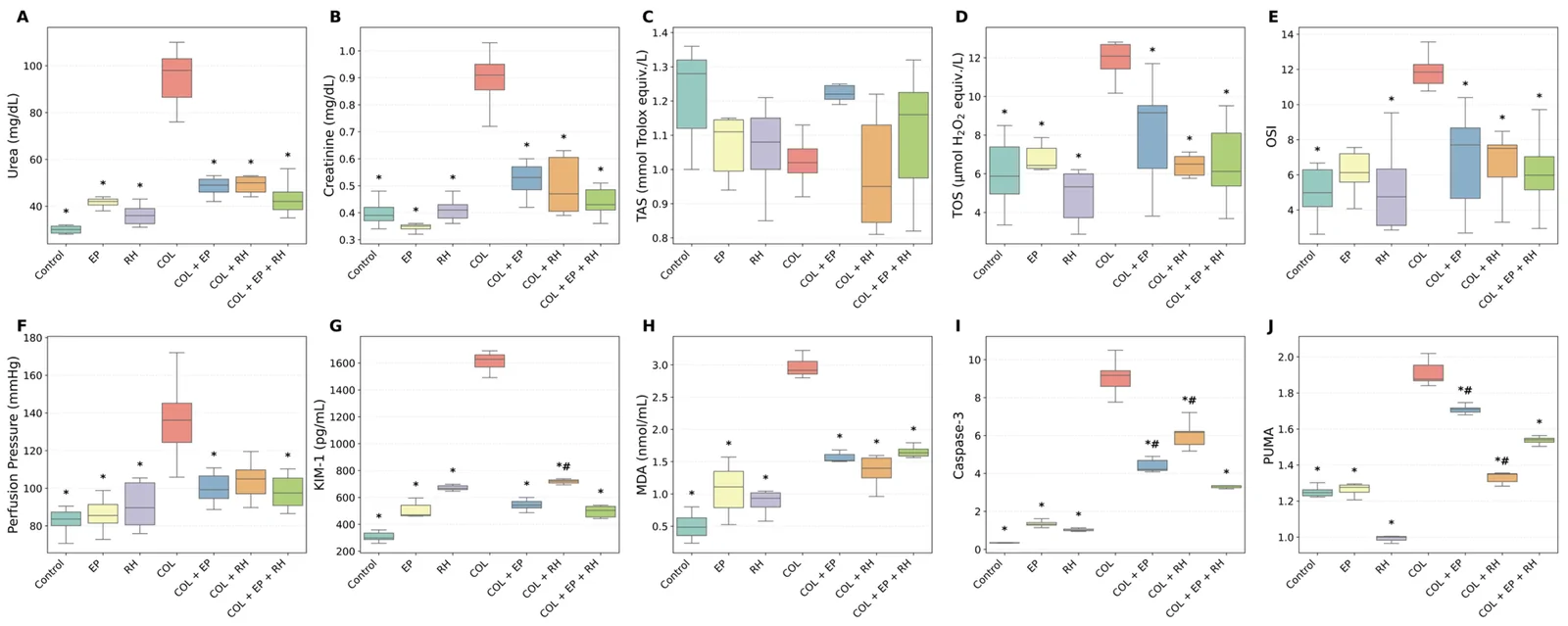
Boxplots showing biochemical, oxidative stress, hemodynamic, and molecular parameters across experimental groups (*n* = 7 per group). Boxes represent the interquartile range (Q1–Q3), horizontal lines indicate the median, and whiskers extend to the minimum and maximum values within 1.5 × IQR. (**A**) Urea, (**B**) Creatinine, (**C**) TAS, (**D**) TOS, (**E**) OSI, (**F**) Perfusion Pressure, (**G**) KIM-1, (**H**) MDA, (**I**) Caspase-3, (**J**) PUMA. * *p* < 0.05 vs. COL group; # *p* < 0.05, vs. the combined treatment group (COL + EP + RH). The # symbol is shown only on the monotherapy groups (COL + EP and COL + RH) to indicate significant differences from the combined treatment group. Statistical analysis was performed using the Kruskal–Wallis test with Mann–Whitney U post-hoc comparisons and Bonferroni correction. COL, colistin; EP, ethyl pyruvate; RH, rutin hydrate; TAS, total antioxidant status; TOS, total oxidant status; OSI, oxidative stress index; KIM-1, kidney injury molecule-1; MDA, malondialdehyde; PUMA, p53 upregulated modulator of apoptosis.

A statistically significant difference was observed among the groups in urea and creatinine levels (*p* < 0.001). Post hoc pairwise comparisons revealed that both parameters were significantly higher in the COL group than in all other groups, and significantly lower in all treatment groups than in the COL group (*p* < 0.05) (Figure 1A,B). Although a significant difference was also found among the groups in total antioxidant status (TAS) values (*p* = 0.037), pairwise analyses did not reveal any statistically significant differences between the individual groups (*p* > 0.05) (Figure 1C). Total oxidant status (TOS) levels were significantly higher in the COL group than in all other groups and significantly lower in all treatment groups than in the COL group (*p* < 0.05) (Figure 1D). Oxidative stress index (OSI) values were also higher in the COL group, with the difference reaching statistical significance against all groups except EP, and were significantly lower in all treatment groups than in the COL group (Figure 1E). Among the treatment groups, urea, creatinine, TOS, and OSI values were numerically lowest in the combined treatment group (COL + EP + RH); however, none of these differences reached statistical significance compared with the single-agent groups (*p* > 0.05) (Table 1).

### 2.2. Perfusion Pressure

Assessment using the Langendorff perfusion system revealed significant differences in perfusion pressures among the groups (*p* < 0.001). In pairwise comparisons, no significant difference was found between the COL and COL + RH groups (*p* > 0.05). However, the COL group was significantly different from all remaining groups (*p* < 0.05), exhibiting higher perfusion pressure compared with the control groups, while treatment groups showed decreased perfusion pressures compared with the COL group. However, in post hoc pairwise comparisons, no significant difference was found between the single treatment groups (COL + EP and COL + RH) and the combined treatment group (COL + EP + RH) (*p* > 0.05) (Figure 1F; Table 1).

### 2.3. Tissue-Level Biomarkers

**Figure 2:**
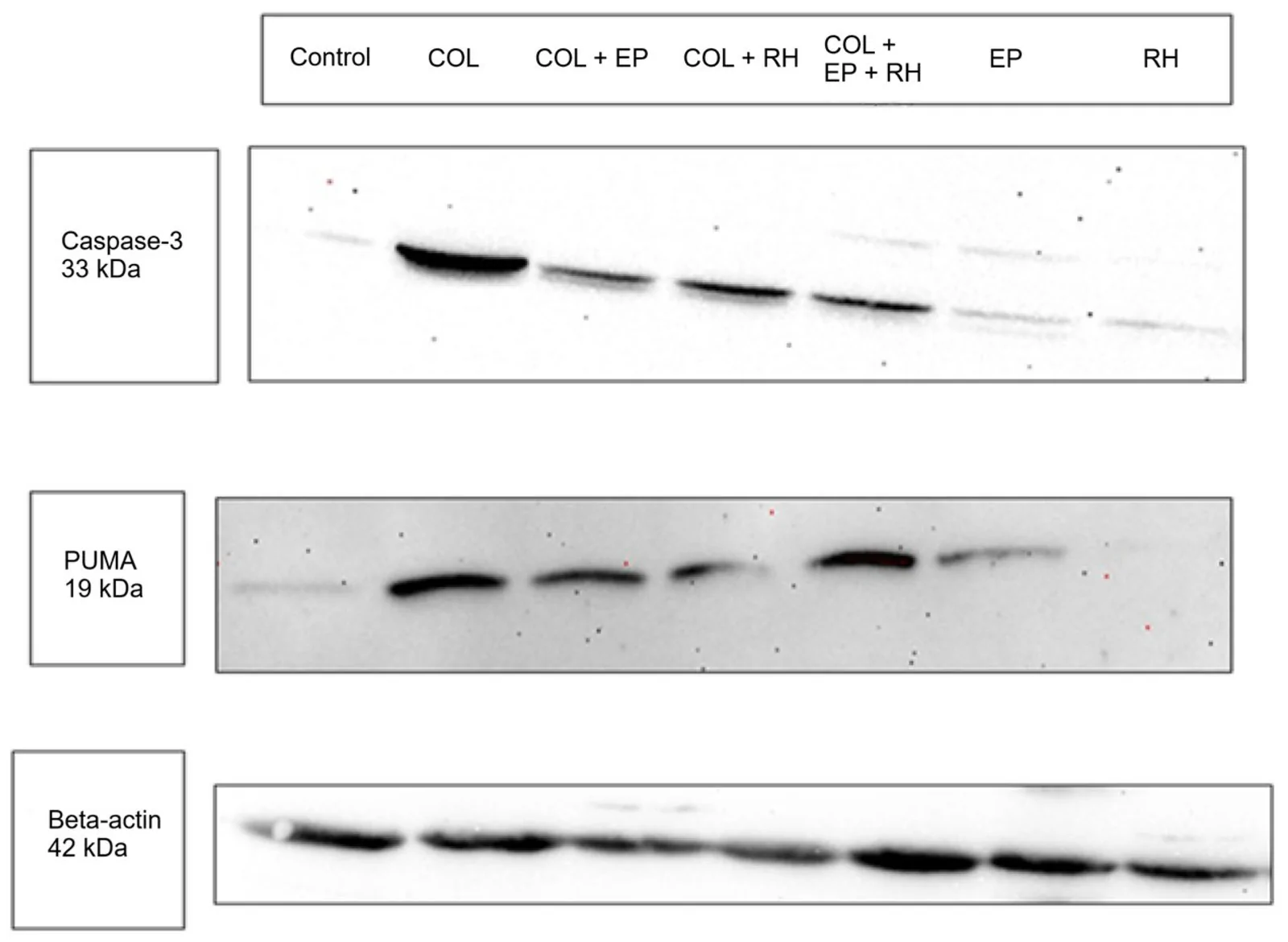
Representative Western blot analysis of caspase-3 and PUMA protein expression demonstrating the protective effects of EP and RH against COL-induced nephrotoxicity in rat kidney tissues (*n* = 7 per group). β-actin was used as the loading control. COL, colistin; EP, ethyl pyruvate; RH, rutin hydrate; PUMA, p53 upregulated modulator of apoptosis. The original uncropped Western blot images used to generate this figure are provided in the Supplementary Materials.

Significant differences were found among the groups in terms of KIM-1 (Figure 1G), MDA (Figure 1H), caspase-3 (Figure 1I), and PUMA (Figure 1J) levels. All biomarker levels were significantly higher in the COL group compared with all other groups (*p* < 0.05). In post hoc pairwise comparisons among the treatment groups, KIM-1 levels differed significantly only between the COL + RH and the combined treatment groups, whereas the difference between the COL + EP and the combined treatment groups was not significant. No significant differences in MDA levels were observed between either single-agent group and the combined treatment group. Caspase-3 levels were significantly lower in the combined treatment group than in both single-agent groups, whereas PUMA levels in the combined treatment group were significantly lower than in the COL + EP group but significantly higher than in the COL + RH group (*p* < 0.05) (Table 1; Figure 2).

### 2.4. Histopathological Examination

**Figure 3:**
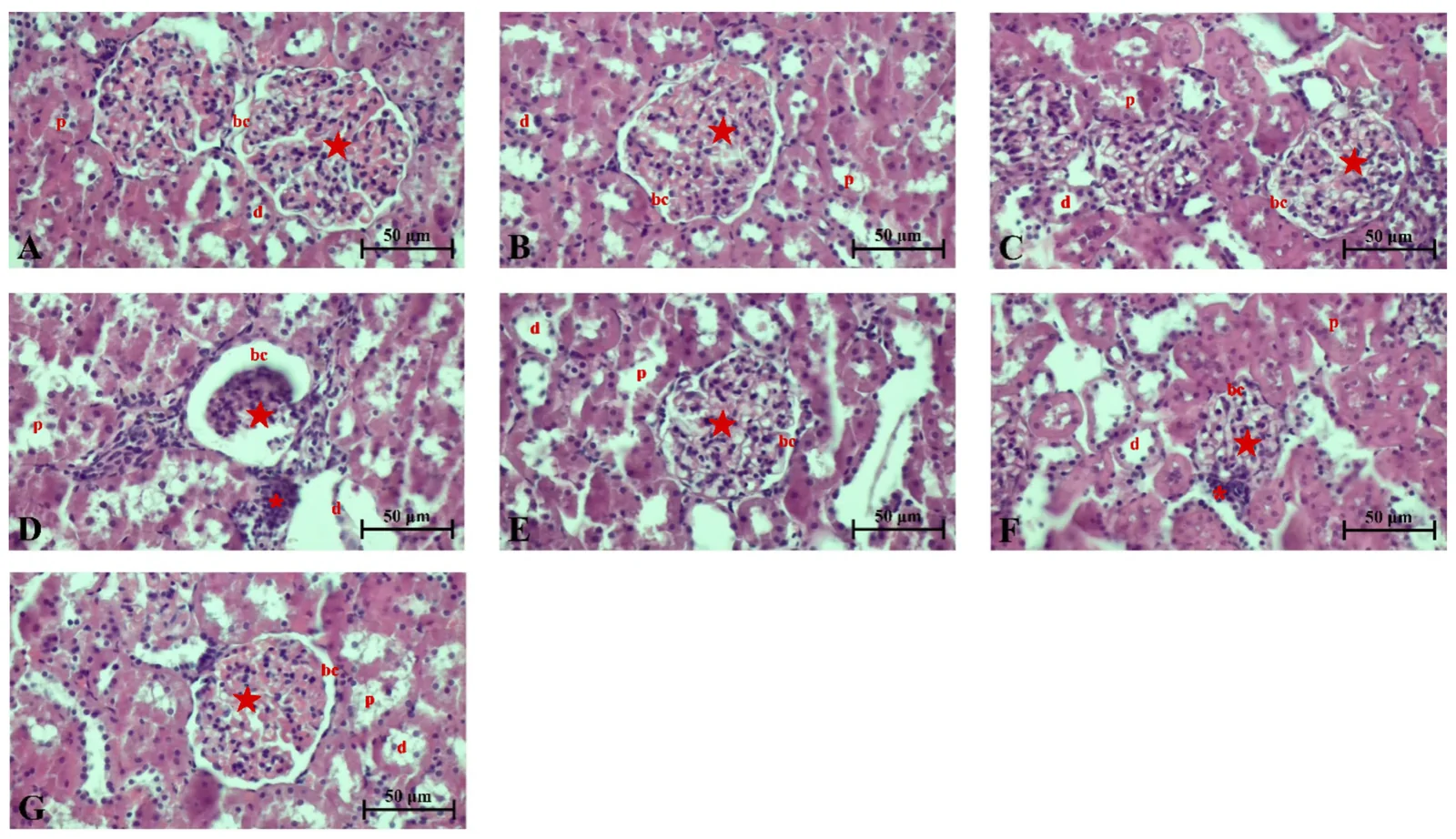
Representative hematoxylin and eosin (H&E) stained renal tissue sections from experimental groups. (**A**) Control, (**B**) EP, (**C**) RH, (**D**) COL, (**E**) COL + EP, (**F**) COL + RH, (**G**) COL + EP + RH. Control, EP, and RH groups showed normal renal histology with intact glomeruli and well-preserved proximal and distal tubular structures. The COL group demonstrated severe histopathological alterations, including tubular epithelial degeneration, tubular necrosis, and desquamation, accompanied by marked leukocyte infiltration (*) and widening of Bowman’s capsule space. Single-agent treatment groups (COL + EP and COL + RH) showed partial improvement, with reduced tubular damage and decreased inflammatory cell infiltration. The combined treatment group (COL + EP + RH) exhibited near-normal renal architecture, with preserved tubular integrity and minimal inflammatory changes. d, distal tubule; p, proximal tubule; bc, Bowman’s capsule; ★, glomerulus; *, leukocyte infiltration. Scale bar = 50 µm. COL, colistin; EP, ethyl pyruvate; RH, rutin hydrate.

Histopathological evaluation following hematoxylin–eosin staining revealed no pathological findings in the control, EP, or RH groups. In contrast, the COL group exhibited glomerular atrophy and narrowing of capillary lumens. Widening of Bowman’s capsule space, degeneration and desquamation of tubular epithelial cells, vacuolization, necrosis, and leukocyte infiltration in the interstitial area were also observed (Figure 3).

In the COL + EP group, the glomeruli retained normal morphology, and although focal areas of tubular degeneration were present, most tubular structures showed signs of epithelial recovery. A reduction in leukocyte infiltration and narrowing of Bowman’s capsule space were noted. In the COL + RH group, histopathological damage was attenuated; however, the attenuation of histopathological alterations was less pronounced than in the COL + EP group. In this group, although the glomeruli exhibited a normal appearance, their size was reduced. Compared with the COL group, evidence of epithelial restoration was observed in both proximal and distal tubules. Leukocyte infiltration in the interstitium was decreased, and the Bowman’s capsule space was narrower. Histopathological lesions were less severe in the COL + EP + RH group, with better-preserved histological architecture than in either single-agent treatment group. Bowman’s capsule space was narrower, the glomerular architecture was preserved, and the tubular structures were intact with well-defined lumens. No remarkable pathological alterations were detected in the interstitial tissue (Figure 3).

**Figure 4:**
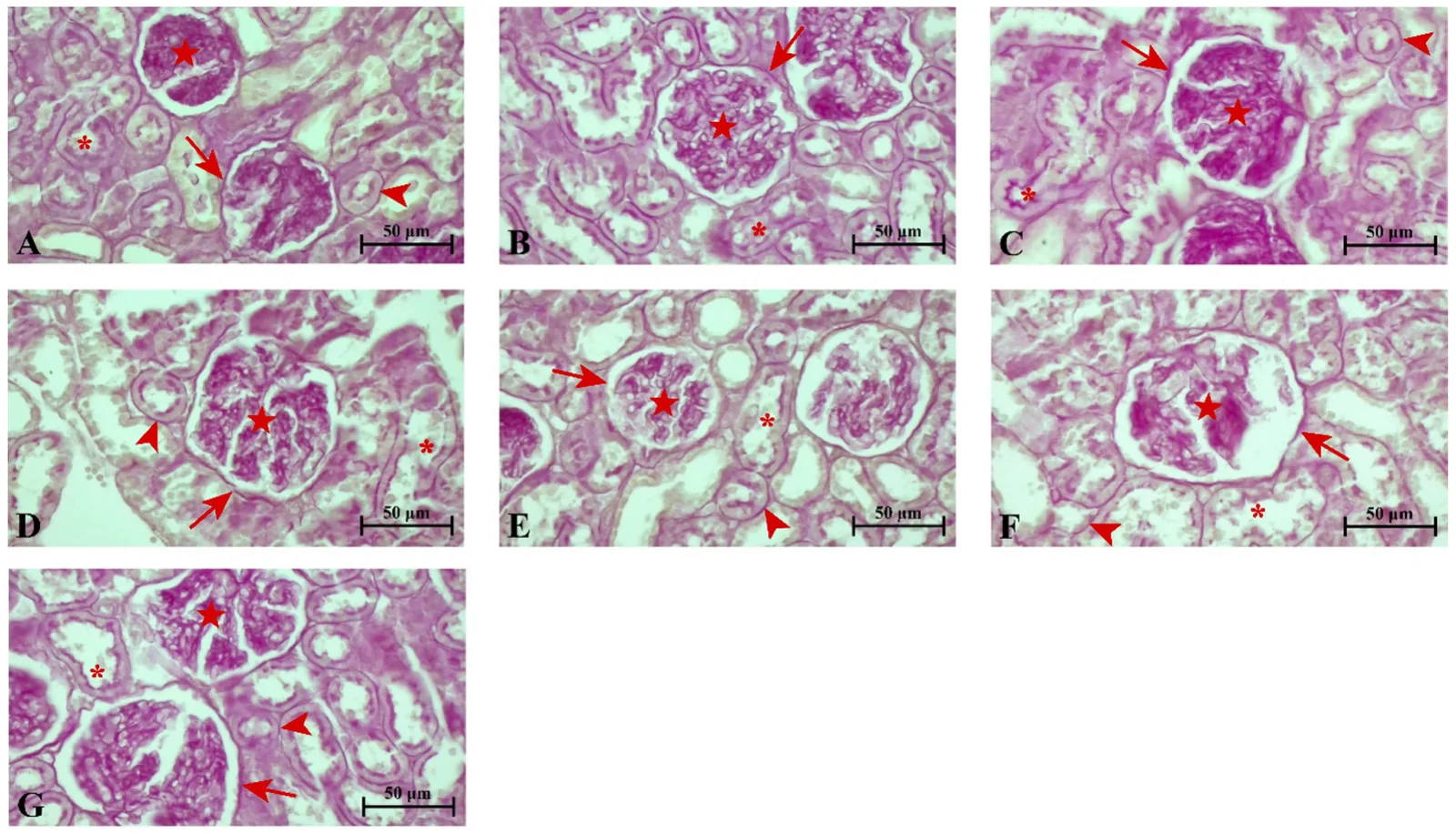
Representative periodic acid–Schiff (PAS) stained renal tissue sections from experimental groups. (**A**) Control, (**B**) EP, (**C**) RH, (**D**) COL, (**E**) COL + EP, (**F**) COL + RH, (**G**) COL + EP + RH. Control, EP, and RH groups exhibited strong PAS positivity in the glomerular basement membrane (GBM), Bowman’s capsule membrane (arrows), and tubular basement membranes, with preserved brush border integrity in proximal tubules. The COL group showed marked histopathological alterations, including thinning and irregularity of the GBM, loss of PAS-positive brush borders in proximal tubules (*), and thinning of distal tubular basement membranes (arrowheads). In the single-agent treatment groups (COL + EP and COL + RH), PAS positivity was partially restored, with improved integrity of the GBM and tubular basement membranes. The combined treatment group (COL + EP + RH) demonstrated near-complete restoration of PAS staining patterns, with well-preserved GBM structure and proximal tubular brush borders. *: proximal tubular lumen; ★, glomerulus; arrows: Bowman’s capsule membrane; arrowheads: distal tubular basement membrane. Scale bar = 50 µm. COL, colistin; EP, ethyl pyruvate; RH, rutin hydrate; GBM, glomerular basement membrane.

Following PAS staining, no pathological changes were identified in the control, EP, or RH groups. In the COL group, thinning and irregularities of the glomerular basement membrane (GBM), loss of PAS-positive brush borders in proximal tubules, and thinning of the distal tubular basement membrane were observed. In the COL + EP and COL + RH groups, compared with the COL group, stronger PAS positivity was noted in the glomerular contents, Bowman’s capsule membrane, and proximal and distal tubular basement membranes, with the improvement being more pronounced in the COL + EP group. In the combined treatment group, PAS staining patterns were closest to those of the control group, with the most complete restoration of the GBM and proximal tubular brush borders (Figure 4).

## 3. Discussion

Antimicrobial resistance remains one of the most pressing global health challenges, driven by extensive antibiotic use and the limited availability of new antimicrobial agents [16,17]. In this context, colistin has re-emerged as a last-resort option for the treatment of multidrug-resistant Gram-negative infections, despite its well-recognized nephrotoxic potential that previously limited its clinical use [3].

Following glomerular filtration, colistin is extensively reabsorbed by proximal tubular cells and accumulates within them. This intracellular accumulation has been proposed as a precondition for colistin-mediated renal damage. Its polycationic nature promotes interaction with anionic membrane phospholipids and increases membrane permeability, and mitochondria have also been suggested as a primary site of injury [18]. Reported consequences include excessive reactive oxygen species generation, lipid peroxidation, depletion of endogenous antioxidant defenses, and an accompanying inflammatory response [19,20]. Apoptotic signaling has been described through mitochondrial, death receptor, and endoplasmic reticulum pathways, which converge on effector caspases such as caspase-3 and culminate in tubular epithelial cell death [18,19]. Oxidative stress, organelle dysfunction, inflammation, and apoptosis therefore appear closely interconnected in colistin-induced nephrotoxicity.

Ethyl pyruvate and rutin may counteract oxidative and apoptotic injury through their antioxidant and cytoprotective properties. Ethyl pyruvate is a stable pyruvate derivative that scavenges reactive oxygen species and supports cellular redox balance. It reduced serum urea, creatinine, and renal malondialdehyde levels in cisplatin-induced nephrotoxicity [21], and attenuated albuminuria and glomerular oxidative stress in diabetic nephropathy [22]. Rutin is a flavonoid glycoside with free-radical-scavenging and metal-chelating properties. It reduced serum urea, creatinine, and lipid peroxidation in hexachlorobutadiene-induced nephrotoxicity [23], and increased renal glutathione while decreasing malondialdehyde in potassium dichromate-induced acute renal injury [24]. Across these models, both agents limited oxidative injury while preserving renal function and structure.

In the present study, the COL group showed significantly higher serum urea and creatinine levels than the control and treatment groups, with creatinine reaching approximately 2.3-fold the control value, indicating successful induction of colistin-associated renal injury, as corroborated by histopathological examination. In the treatment groups, these elevations were markedly attenuated, supporting a renoprotective effect of EP and RH against colistin-induced renal injury. Serum urea and creatinine levels were numerically lowest in the combined treatment group (COL + EP + RH), but the differences from the single-agent co-treatment groups were not statistically significant (*p* > 0.05).

In parallel with the changes in urea and creatinine, TOS and OSI were significantly elevated in the COL group and significantly reduced in all treatment groups. TAS, however, showed no significant pairwise differences. Similar heterogeneity has been reported in comparable nephrotoxicity models, where TAS has been found unchanged, increased, or treatment-dependent [25,26,27]. The TAS results were therefore not interpreted as evidence of a treatment effect. Because TAS reflects the combined activity of many antioxidants, opposing changes in individual components can leave the overall capacity largely unchanged [28]. TOS measures oxidant status itself, and OSI expresses oxidant burden relative to antioxidant capacity [29]. The changes in these two parameters therefore give a more consistent picture, indicating that colistin shifted the redox balance toward oxidation and that EP and RH attenuated this shift.

The perfusion pressure (PP) measurements obtained via the Langendorff system revealed a significant increase in the COL group. This elevation indicates impaired renal perfusion, suggesting that nephrotoxicity manifests not only at the biochemical level but also at the physiological level. Similarly, in experimental nephrotoxicity models induced by cisplatin, studies utilizing the Langendorff system reported elevated PP values in the cisplatin-treated groups, which significantly decreased following therapeutic interventions [21,30]. In the present study, a general trend toward decreased PP values was observed in the treatment groups. Notably, PP levels were significantly reduced in the COL + EP and COL + EP + RH groups, suggesting that both therapeutic agents may play a protective role against colistin-induced vascular effects. Although a marked decrease in PP was also observed in the COL + RH group, the difference compared with the COL group did not reach statistical significance.

KIM-1 is overexpressed following acute tubular injury and has been proposed as a more reliable early marker of colistin-associated nephrotoxicity than serum creatinine, which may reflect relatively advanced renal damage [18]. In prior work on colistin nephrotoxicity investigating the protective effects of dexmedetomidine and an α-helical amphipathic peptide, KIM-1 levels were significantly elevated in the colistin-administered groups, whereas significant reductions were observed in the treatment groups [27,31]. Similarly, in the present study, KIM-1 levels were significantly elevated in the COL group compared with the control groups and significantly reduced in all co-treatment groups relative to the COL group. Among the co-treatment groups, KIM-1 levels were lowest in the COL + EP + RH group; however, they were significantly lower than those in the COL + RH group but not significantly different from those in the COL + EP group. These findings support the protective effects of EP and RH against colistin-associated tubular injury.

In studies evaluating various protective agents against colistin-induced nephrotoxicity, MDA levels were reported to be elevated in colistin-treated groups compared with those in controls, whereas decreased levels were observed in treatment groups [20,32]. In the present study, MDA levels were also significantly greater in the COL group than in the control groups, whereas statistically significant decreases were observed in the treatment groups. These findings support the notion that the antioxidant properties of EP and RH are effective in mitigating colistin-induced oxidative stress.

Caspase-3 levels were significantly higher in the COL group than in the control groups and significantly lower in all treatment groups than in the COL group, consistent with previous studies evaluating alpha-lipoic acid and corynoxeine in colistin-induced nephrotoxicity [33,34]. The lowest caspase-3 level was observed in the combined treatment group (COL + EP + RH) and was significantly lower than that in both single-agent co-treatment groups.

In previous cisplatin-induced nephrotoxicity models in which pifithrin-α and fenofibrate were used as protective agents, PUMA levels were reported to be significantly increased in the cisplatin-treated groups compared with those in the control groups, but decreased in the treatment groups compared with those in the cisplatin group [35,36]. Similarly, in the present study, PUMA levels were significantly elevated in the COL group compared with the control groups and significantly reduced in all treatment groups. The lowest values were observed in the COL + RH group, which may indicate a stronger effect of RH on this early apoptotic mediator. Values in the combined treatment group were significantly lower than in the COL + EP group but significantly higher than in the COL + RH group, so the combination did not show a uniform advantage across the apoptotic markers.

Previous studies investigating colistin-induced nephrotoxicity have reported histopathological findings such as tubular dilatation, detachment from surrounding tissue, cast formation, and epithelial cell degeneration and regeneration [37]. Another study reported that exposure to colistin caused significant tubular damage; this damage was characterized by focal necrosis of tubular epithelial cells, formation of cellular and proteinaceous casts, and signs of acute tubular necrosis [38]. Similarly, in an experimental study, colistin administration led to focal tubular dilatation, vacuolar degeneration, loss of brush borders, and tubular epithelial desquamation, while these alterations improved in the treatment group [39]. Consistent with previous models, this study revealed that colistin administration induces pronounced renal injury characterized by glomerular atrophy, narrowing of the capillary lumens, widening of Bowman’s capsule space, tubular degeneration with vacuolization and necrosis, and interstitial leukocyte infiltration. PAS staining further demonstrated glomerular basement membrane irregularities and loss of proximal tubular brush borders. Compared with colistin alone, both EP and RH mitigated these lesions. On qualitative assessment, EP produced the most evident histological recovery, with reduced tubular epithelial damage, decreased leukocyte infiltration, and preservation of GBM and proximal tubular integrity on PAS. RH provided meaningful but comparatively smaller improvements. Notably, the combined treatment group (COL + EP + RH) showed near-normal glomerular and tubular architecture with minimal interstitial inflammation, in line with the protective effects observed at the biochemical and molecular levels.

Taken together, these findings suggest a biologically plausible, although unproven, link between oxidative stress and tubular apoptosis in colistin-induced nephrotoxicity. Colistin-associated mitochondrial dysfunction and excessive reactive oxygen species generation [18,19] are consistent with the higher TOS, OSI, and renal MDA levels observed in the COL group. Oxidative stress may, in turn, engage p53-dependent PUMA signaling and downstream caspase-3 activation [36,40], in line with the concurrent increases in PUMA and caspase-3 in the COL group and their reductions in the treatment groups. KIM-1, which has been shown to increase in colistin-induced proximal tubular and brush-border injury [41], was likewise elevated, while histopathological examination demonstrated tubular degeneration, necrosis, and loss of the brush border. The parallel reductions in TOS, OSI, MDA, PUMA, caspase-3, and KIM-1, together with the histological improvement, indicate that EP and RH may attenuate interconnected components of colistin-induced renal injury. However, because mitochondrial function and intermediate signaling events were not directly assessed, these relationships should be interpreted as associations rather than evidence of causality, and further mechanistic studies are needed.

A particular strength of this study is that it is the first to evaluate the protective effects of ethyl pyruvate and rutin hydrate, alone and in combination, against colistin-induced nephrotoxicity and that the resulting renal injury was characterized comprehensively at the biochemical, physiological, molecular, and histopathological levels, including direct assessment of renal hemodynamics in an isolated perfused kidney preparation. Nevertheless, several limitations should be acknowledged. The study was conducted using only a male Wistar albino rat model; therefore, the findings may not fully reflect sex-related differences or human renal physiology, limiting their direct generalization to clinical settings. The group size, although exceeding the minimum indicated by the resource equation, provided adequate power only for large effects, which may partly explain why the differences between the combined and single-agent treatments did not reach statistical significance for several parameters. In addition, colistin was administered for only seven days, precluding the evaluation of long-term or chronic effects, and all agents were given at fixed doses via the intraperitoneal route, so the influence of different doses or administration routes was not assessed. Moreover, because all parameters were assessed only at the end of the experimental period, their temporal patterns and potential group-specific trajectories could not be characterized. Future studies in animals of both sexes, incorporating dose–response analyses, alternative administration routes, longer treatment durations, and assessments at multiple time points, are warranted to confirm and extend these findings. Whether combined administration provides additional benefit over either agent alone remains to be determined in studies powered for such comparisons.

## 4. Materials and Methods

### 4.1. Study Design and Setting

In this study, a nephrotoxicity model was established in rat kidneys via colistin, and the potential protective effects of ethyl pyruvate and rutin hydrate were evaluated.

A total of 49 adult male Wistar albino rats were allocated to seven experimental groups, with seven rats in each group, by haphazard selection from the holding cages. The experimental unit was the individual animal. Inclusion criteria were healthy, experimentally naive male Wistar albino rats weighing 250–300 g, obtained from the Dicle University Health Sciences Research and Application Center (DÜSAM), Diyarbakır, Türkiye. Throughout the experiment, the rats were housed under standard laboratory conditions at a temperature of 23 ± 2 °C, a relative humidity of 60%, and a 12-h light/12-h dark cycle. All the animals had ad libitum access to standard laboratory feed and water. As the animals were bred and maintained in the same facility under these conditions, no separate acclimatization period was required. Animal health was monitored daily by weighing and by recording food and water intake. A body-weight loss exceeding 15%, or signs of illness or injury, was defined a priori as both a humane endpoint and an exclusion criterion; no animal reached this endpoint, no adverse events or unexpected deaths occurred, and no animals or data points were excluded from any analysis. The care and monitoring of the animals were conducted in strict accordance with the ethical principles outlined in the Guide for the Care and Use of Laboratory Animals [42].

Throughout the study, the term “control group” refers specifically to the vehicle-treated group that received only the solvents without any pharmacological agents, whereas the term “control groups” collectively refers to the vehicle, EP, and RH groups, all of which did not receive colistin and therefore served as controls.

Each animal in every experimental group received a total daily intraperitoneal (i.p.) injection volume of 3 mL, consisting of the respective solvents with or without the assigned pharmacological agents. Colistin was dissolved in normal saline and administered at 5 mg/kg once daily for seven days, resulting in a cumulative dose of 35 mg/kg, which is comparable to the cumulative dose of 36.5 mg/kg previously shown to induce biochemical and histopathological renal injury in rats [43]. EP and RH were dissolved in Ringer’s lactate solution and dimethyl sulfoxide, respectively, and each was given once daily at 50 mg/kg. These doses were based on previous rodent studies demonstrating the efficacy of EP, including in a renal injury model [21,44], and of RH in rats [45]. All injections were performed by the same investigator at approximately the same time of day (Table 2). In groups receiving more than one agent, colistin and the protective agent(s) were given as separate injections within the same daily dosing session. The protective agents were initiated on day 1 concurrently with colistin rather than after renal injury had been established. Because only a limited number of animals could be processed on a single day, treatment was initiated in a staggered manner; nevertheless, every animal received the same seven-day protocol and was sacrificed on day 8 of its own schedule. All animals were housed in the same room under identical environmental conditions, and all samples designated for biochemical and molecular analyses were stored at −80 °C and analyzed in a single batch.

On the eighth day of the experiment, the rats were sacrificed. Anesthesia was induced via a combination of ketamine (85 mg/kg, i.m.) and xylazine (15 mg/kg, i.m.) [21]. The depth of anesthesia was confirmed by the absence of the pedal withdrawal reflex to toe pinch before the first incision, and heparinization was then performed. A midline incision was subsequently made to perform a laparotomy. The left kidney was carefully dissected from surrounding tissues, and the renal artery was anatomically identified and marked with a silk suture to preserve its orientation. The kidney was then excised, immediately placed in prewarmed Krebs solution at 37 °C, and continuously aerated with 95% O_2_ and 5% CO_2_. After placement in a Petri dish, the renal artery was cannulated via a yellow-tipped angiocatheter and secured with a prepositioned silk suture.

The kidney was then connected to a Langendorff perfusion system, to allow direct assessment of renal vascular function, containing Krebs solution maintained at 37 °C and continuously aerated with 95% O_2_ and 5% CO_2_, and delivered at a constant flow rate of 7 mL/min. The perfusion pressure was monitored for approximately 60 min using a Biopac MP30 data acquisition system (Biopac Systems Inc., Santa Barbara, CA, USA).

The other (right) kidney was excised via a similar surgical procedure, divided into three portions, and stored for subsequent analyses. Animals remained under deep anesthesia throughout and were sacrificed by exsanguination during cardiac blood collection, without regaining consciousness.

### 4.2. Collection of Blood Samples

Following the removal of the kidneys, a thoracotomy was performed, and approximately 5 mL of blood was collected directly from the heart. The blood samples were centrifuged at 4000 rpm for 15 min, and the obtained serum was stored at −80 °C for later analyses. In the serum samples, urea and creatinine levels, total antioxidant status (TAS) [28], and total oxidant status (TOS) [29] (Rel Assay Diagnostic Kits, Mega Tip, Gaziantep, Türkiye) were measured, and the oxidative stress index (OSI) was calculated as OSI (arbitrary unit) = TOS (μmol H_2_O_2_ equiv./L)/TAS (mmol Trolox equiv./L). As no standardized reference value exists for OSI, it was used only for between-group comparisons [46].

### 4.3. ELISA and Western Blotting

For the ELISA analyses, the levels of kidney injury molecule-1 (KIM-1) and malondialdehyde (MDA) were measured in the supernatant samples prepared from kidney tissues. KIM-1 concentrations were determined via the Rat KIM-1 ELISA Kit (SunRed, Cat. No: 201-11-0550, Shanghai, China), and MDA levels were quantified via the Rat MDA ELISA Kit (BT Lab, Cat. No: E0156Ra, Shanghai, China) following the manufacturers’ protocols. Standard curves for both biomarkers were generated via a four-parameter logistic (4PL) model, and the optical density was measured at 450 nm. The results were expressed as pg/mL (KIM-1) and nmol/mL (MDA).

For Western blot analysis, total proteins isolated from kidney tissues were separated by SDS-PAGE and transferred onto PVDF membranes. The membranes were incubated with primary antibodies against p53 upregulated modulator of apoptosis (PUMA) (Cat. No. ab9643, Abcam, Cambridge, UK) and cysteine-aspartic acid protease-3 (caspase-3) (19677-1-AP, Proteintech, Rosemont, IL, USA), followed by incubation with HRP-conjugated secondary antibodies. The resulting chemiluminescent bands were visualized using an iBright™ Imaging System (Thermo Fisher Scientific, Waltham, MA, USA). Band intensities were quantified using ImageJ software (version 1.53e; National Institutes of Health, Bethesda, MD, USA), and the results were normalized to β-actin as the reference protein.

### 4.4. Histopathological Examination

Kidney tissues were evaluated histologically using hematoxylin–eosin (H&E) and periodic acid–Schiff (PAS) staining. Sections were examined under a light microscope at ×20 magnification by two independent histologists blinded to the experimental groups. Each section was assessed for both glomerular and tubulointerstitial changes. Glomerular evaluation included glomerular atrophy, narrowing of the capillary lumens, widening of Bowman’s capsule space, and thinning or irregularity of the glomerular basement membrane. At the tubular level, we recorded epithelial degeneration, desquamation of tubular epithelial cells, cytoplasmic vacuolization, tubular necrosis, loss of the PAS-positive brush border in the proximal tubules, and thinning of the tubular basement membranes. Interstitial changes were assessed on the basis of inflammatory (leukocyte) cell infiltration.

### 4.5. Statistical Analysis

Since none of the continuous variables followed a normal distribution (assessed by the Shapiro–Wilk test), all data are presented as medians and 25th–75th percentiles (Q1–Q3). Differences among groups were assessed using the nonparametric Kruskal–Wallis test. For variables showing significant differences, post hoc pairwise group comparisons were conducted via the Mann–Whitney U test with Bonferroni correction. Group size was determined a priori using the resource equation approach [47], which indicates three animals per group for a seven-group design (error degrees of freedom = 14). Seven animals per group were used to allow for possible losses and to remain consistent with group sizes used in comparable rodent studies of drug-induced nephrotoxicity [20,32,33].

A *p*-value of <0.05 was considered statistically significant in all analyses. All data processing, statistical analyses, and graphical visualizations were performed via the Python programming language (version 3.11) and its relevant statistical and visualization libraries.

## 5. Conclusions

This experimental study successfully established a colistin-induced nephrotoxicity model at the biochemical, physiological, molecular, and histopathological levels and demonstrated that the resulting renal injury was significantly reduced by once-daily intraperitoneal administration of EP (50 mg/kg) and RH (50 mg/kg), alone or in combination, for seven consecutive days. These findings indicate that both agents exert antioxidant, antiapoptotic, and nephroprotective effects, with their combined administration yielding the numerically greatest overall benefit. Collectively, these results suggest that EP and RH may constitute promising candidate agents to attenuate colistin-induced nephrotoxicity.

## Figures and Tables

**Table 1:** Distribution of parameters demonstrating the effects of EP and RH on COL-induced nephrotoxicity (median, (Q1–Q3)).

Groups ^1,2^	Urea (mg/dL) ^3^	Creatinine (mg/dL) ^4^	TAS (mmol Trolox equiv./L) ^5^	TOS (μmol H_2_O_2_ equiv./L) ^6^	OSI (a.u.) ^7^
Control	30 (28.5–31.5)	0.39 (0.37–0.42)	1.28 (1.12–1.32)	5.87 (4.96–7.38)	4.98 (4.18–6.29)
EP	42 (40.5–43)	0.35 (0.34–0.35)	1.11 (0.99–1.15)	6.43 (6.28–7.32)	6.12 (5.58–7.20)
RH	36 (32.5–39)	0.41 (0.38–0.43)	1.08 (1.00–1.15)	5.32 (3.73–5.99)	4.75 (3.12–6.32)
COL	98 (86.5–103)	0.91 (0.85–0.95)	1.02 (0.99–1.06)	12.08 (11.43–12.69)	11.84 (11.20–12.28)
COL + EP	49 (46–51.5)	0.53 (0.48–0.57)	1.22 (1.21–1.25)	9.16 (6.28–9.53)	7.70 (4.66–8.67)
COL + RH	50 (46–52.5)	0.47 (0.41–0.60)	0.95 (0.84–1.13)	6.50 (5.92–6.89)	7.51 (5.87–7.70)
COL + EP + RH	42 (38.5–46)	0.43 (0.41–0.48)	1.16 (0.98–1.23)	6.12 (5.37–8.10)	5.98 (5.14–7.03)
**Groups**	**PP (mmHg) ^8^**	**KIM-1 (pg/mL) ^9^**	**MDA (nmol/mL) ^10^**	**Caspase-3 ^11^**	**PUMA ^12^**
Control	83.60 (80.10–87.35)	298.35 (286.60–334.55)	0.48 (0.36–0.63)	0.35 (0.33–0.36)	1.25 (1.23–1.26)
EP	85.50 (81.50–91.35)	470.08 (463.27–541.57)	1.11 (0.79–1.35)	1.33 (1.27–1.41)	1.28 (1.25–1.29)
RH	89.60 (80.55–102.85)	666.85 (657.89–686.81)	0.93 (0.80–1.02)	1.00 (0.99–1.06)	1.00 (0.98–1.00)
COL	136.10 (124.32–145.15)	1627.28 (1569.97–1660.85)	2.92 (2.86–3.05)	9.17 (8.59–9.41)	1.88 (1.87–1.95)
COL + EP	99.20 (94.57–106.53)	541.97 (521.86–569.46)	1.52 (1.51–1.61)	4.22 (4.16–4.69)	1.71 (1.69–1.72)
COL + RH	104.90 (97.05–109.70)	720.93 (706.90–730.00)	1.40 (1.25–1.56)	6.16 (5.53–6.22)	1.35 (1.31–1.35)
COL + EP + RH	97.50 (90.80–105.37)	502.88 (454.16–533.04)	1.64 (1.59–1.69)	3.35 (3.25–3.35)	1.54 (1.53–1.55)

^1^ Values are presented as median (Q1–Q3); *n* = 7 per group. Pairwise comparisons were performed using the Mann–Whitney U test with Bonferroni correction following significant Kruskal–Wallis results. A *p*-value < 0.05 was considered statistically significant. ^2^ Kruskal–Wallis test results: Urea, *p* < 0.001; Creatinine, *p* < 0.001; TAS, *p* = 0.037; TOS, *p* < 0.01; OSI, *p* < 0.01; PP, *p* < 0.001; KIM-1, *p* < 0.001; MDA, *p* < 0.001; Caspase-3, *p* < 0.001; PUMA, *p* < 0.001. ^3^ Urea: COL vs. all groups, *p* < 0.05. ^4^ Creatinine: COL vs. all groups, *p* < 0.05. ^5^ TAS: COL vs. all groups, *p* > 0.05 (no significant pairwise differences). ^6^ TOS: COL vs. all groups, *p* < 0.05. ^7^ OSI: COL vs. EP, *p* > 0.05; COL vs. all other groups, *p* < 0.05. ^8^ PP: COL vs. COL + RH, *p* > 0.05; COL vs. all other groups, *p* < 0.05. ^9^ KIM-1, ^10^ MDA, ^11^ Caspase-3, ^12^ PUMA (/β-actin (a.u.)): COL vs. all groups, *p* < 0.05. OSI is expressed in arbitrary units (a.u.) and was calculated as TOS (μmol H_2_O_2_ equiv./L)/TAS (mmol Trolox equiv./L). Caspase-3 and PUMA values are expressed as protein band intensities normalized to β-actin (arbitrary units, a.u.). COL, colistin; EP, ethyl pyruvate; RH, rutin hydrate; TAS, total antioxidant status; TOS, total oxidant status; OSI, oxidative stress index; PP, perfusion pressure; KIM-1, kidney injury molecule-1; MDA, malondialdehyde; PUMA, p53 upregulated modulator of apoptosis.

**Table 2:** Experimental groups and administered treatments.

Group	n	Normal Saline (NS)	Ringer’s Lactate (RL)	Dimethyl Sulfoxide (DMSO)	Route of Administration
Control	7	1 mL	1 mL	1 mL	i.p.
Ethyl Pyruvate (EP)	7	1 mL	Ethyl pyruvate (50 mg/kg) in 1 mL	1 mL	i.p.
Rutin Hydrate (RH)	7	1 mL	1 mL	Rutin hydrate (50 mg/kg) in 1 mL	i.p.
Colistin (COL)	7	Colistin (5 mg/kg) in 1 mL	1 mL	1 mL	i.p.
COL + EP	7	Colistin (5 mg/kg) in 1 mL	Ethyl pyruvate (50 mg/kg) in 1 mL	1 mL	i.p.
COL + RH	7	Colistin (5 mg/kg) in 1 mL	1 mL	Rutin hydrate (50 mg/kg) in 1 mL	i.p.
COL + EP + RH	7	Colistin (5 mg/kg) in 1 mL	Ethyl pyruvate (50 mg/kg) in 1 mL	Rutin hydrate (50 mg/kg) in 1 mL	i.p.

## Data Availability

The original contributions presented in this study are included in the article/Supplementary Materials. Further inquiries can be directed to the corresponding author.

## Supplementary Materials

The following supporting information can be downloaded at: https://www.mdpi.com/article/10.3390/ph19091417/s1.

## Abbreviations

The following abbreviations are used in this manuscript:

4PL	four-parameter logistic
a.u.	arbitrary units
caspase-3	cysteine-aspartic acid protease-3
COL	colistin
DMSO	dimethyl sulfoxide
ELISA	enzyme-linked immunosorbent assay
EP	ethyl pyruvate
GBM	glomerular basement membrane
H&E	hematoxylin and eosin
HRP	horseradish peroxidase
i.m.	intramuscular
i.p.	intraperitoneal
KIM-1	kidney injury molecule-1
MDA	malondialdehyde
NS	normal saline
OSI	oxidative stress index
PAS	periodic acid–Schiff
PP	perfusion pressure
PUMA	p53 upregulated modulator of apoptosis
PVDF	polyvinylidene difluoride
RH	rutin hydrate
RL	Ringer’s lactate
SDS-PAGE	sodium dodecyl sulfate–polyacrylamide gel electrophoresis
TAS	total antioxidant status
TOS	total oxidant status

## References

[1] Hutchings M.I., Truman A.W., Wilkinson B. (2019). Antibiotics: Past, present and future. Curr. Opin. Microbiol..

[2] Fleming A. Penicillin Nobel Lecture. https://www.nobelprize.org/prizes/medicine/1945/fleming/lecture/.

[3] Nation R.L., Li J. (2009). Colistin in the 21st century. Curr. Opin. Infect. Dis..

[4] Li P., Li S., Zhang J., Zeng S., Li Z., Xie J., Yang Y. (2025). Factors affecting the effectiveness and safety of colistin in treating drug-resistant gram-negative bacterial infections: A meta-analysis. Front. Pharmacol..

[5] Avedissian S.N., Liu J., Rhodes N.J., Lee A., Pais G.M., Hauser A.R., Scheetz M.H. (2019). A review of the clinical pharmacokinetics of polymyxin B. Antibiotics.

[6] Mirjalili M., Mirzaei E., Vazin A. (2022). Pharmacological agents for the prevention of colistin-induced nephrotoxicity. Eur. J. Med. Res..

[7] Aggarwal R., Dewan A. (2018). Comparison of nephrotoxicity of Colistin with Polymyxin B administered in currently recommended doses: A prospective study. Ann. Clin. Microbiol. Antimicrob..

[8] Wang J., Niu H., Wang R., Cai Y. (2019). Safety and efficacy of colistin alone or in combination in adults with Acinetobacter baumannii infection: A systematic review and meta-analysis. Int. J. Antimicrob. Agents.

[9] Ko H., Jeon M., Choo E., Lee E., Kim T., Jun J.B., Gil H.W. (2011). Early acute kidney injury is a risk factor that predicts mortality in patients treated with colistin. Nephron Clin. Pract..

[10] DeRyke C.A., Crawford A.J., Uddin N., Wallace M.R. (2010). Colistin dosing and nephrotoxicity in a large community teaching hospital. Antimicrob. Agents Chemother..

[11] Fink M.P. (2008). Ethyl pyruvate. Curr. Opin. Anaesthesiol..

[12] Yang R., Zhu S., Tonnessen T.I. (2016). Ethyl pyruvate is a novel anti-inflammatory agent to treat multiple inflammatory organ injuries. J. Inflamm..

[13] Çelik H., Kandemir F.M., Caglayan C., Özdemir S., Çomaklı S., Kucukler S., Yardım A. (2020). Neuroprotective effect of rutin against colistin-induced oxidative stress, inflammation and apoptosis in rat brain associated with the CREB/BDNF expressions. Mol. Biol. Rep..

[14] Ghorbani A. (2017). Mechanisms of antidiabetic effects of flavonoid rutin. Biomed. Pharmacother..

[15] Miklasińska-Majdanik M., Kępa M., Wąsik T.J., Zapletal-Pudełko K., Klim M., Wojtyczka R.D. (2023). The Direction of the Antibacterial Effect of Rutin Hydrate and Amikacin. Antibiotics.

[16] Murray C.J., Ikuta K.S., Sharara F., Swetschinski L., Aguilar G.R., Gray A., Han C., Bisignano C., Rao P., Wool E. (2022). Global burden of bacterial antimicrobial resistance in 2019: A systematic analysis. Lancet.

[17] Appelbaum P.C. (2012). 2012 and beyond: Potential for the start of a second pre-antibiotic era?. J. Antimicrob. Chemother..

[18] Gai Z., Samodelov S.L., Kullak-Ublick G.A., Visentin M. (2019). Molecular mechanisms of colistin-induced nephrotoxicity. Molecules.

[19] Karimzadeh I., Kane-Gill S.L., Ma B. (2025). Anti-infective-associated AKI: A narrative review of the epidemiology, mechanisms, risk factors, biomarkers, clinical course, monitoring, prevention, and therapeutic strategies. Antibiotics.

[20] Edrees N.E., Galal A.A., Monaem A.R.A., Beheiry R.R., Metwally M.M. (2018). Curcumin alleviates colistin-induced nephrotoxicity and neurotoxicity in rats via attenuation of oxidative stress, inflammation and apoptosis. Chem. Biol. Interact..

[21] Kelle I., Akkoc H., Tunik S., Nergiz Y., Erdinc M., Erdinc L. (2014). Protective effects of ethyl pyruvate in cisplatin-induced nephrotoxicity. Biotechnol. Biotechnol. Equip..

[22] Ju K.D., Shin E.K., Cho E.J., Yoon H.B., Kim H.S., Kim H., Yang J., Hwang Y.-H., Ahn C., Oh K.-H. (2012). Ethyl pyruvate ameliorates albuminuria and glomerular injury in the animal model of diabetic nephropathy. Am. J. Physiol.-Ren. Physiol..

[23] Sadeghnia H.R., Yousefsani B.S., Rashidfar M., Boroushaki M.T., Asadpour E., Ghorbani A. (2013). Protective effect of rutin on hexachlorobutadiene-induced nephrotoxicity. Ren. Fail..

[24] Mohsen A.M., Wagdi M.A., Salama A. (2024). Rutin loaded bilosomes for enhancing the oral activity and nephroprotective effects of rutin in potassium dichromate induced acute nephrotoxicity in rats. Sci. Rep..

[25] Erkılınç G., Bedir M., Ayözger L.E.Ö., Doğan H.K., Karakuyu N.F. (2023). Nephroprotective Effect of Astaxanthin Against Radiotherapy Via TAS, TOS (Biochemical), TNF-α, CASPASE-3 (Immunohistochemical), SIRT 1-P53 (Molecular) Pathway in Rats. Genel Tıp Derg..

[26] Tekes E., Ickin Gulen M., Silan C., Guven Bagla A. (2025). Humic acid attenuates cisplatin-induced nephrotoxicity in rats. Drug Chem. Toxicol..

[27] Talih G., Esmaoğlu A., Bayram A., Yazici C., Deniz K., Talih T. (2018). Does dexmedetomidine prevent colistin nephrotoxicity?. Braz. J. Anesthesiol. (Engl. Ed.).

[28] Erel O. (2004). A novel automated direct measurement method for total antioxidant capacity using a new generation, more stable ABTS radical cation. Clin. Biochem..

[29] Erel O. (2005). A new automated colorimetric method for measuring total oxidant status. Clin. Biochem..

[30] Erdinç M., Erdinç L., Nergiz Y., Kelle İ. (2007). The effects of nifedipine on renal perfusion pressure and kidney during cisplatin-induced nephrotoxicity in rats. Dicle Med. J..

[31] Kook M.S., Kim H., Choi Y., Bae S.M., Yu J., Kim Y.S. (2025). The α-Helical Amphipathic Peptide Alleviates Colistin-Induced Nephrotoxicity by Maintaining Mitochondrial Function in Both In Vitro and In Vivo Infection Models. Antibiotics.

[32] Rajabalizadeh R., Rahbardar M.G., Razavi B.M., Hosseinzadeh H. (2024). Renoprotective effects of crocin against colistin-induced nephrotoxicity in a rat model. Iran. J. Basic Med. Sci..

[33] Oktan M.A., Heybeli C., Ural C., Kocak A., Bilici G., Cavdar Z., Ozbal S., Arslan S., Yilmaz O., Cavdar C. (2021). Alpha-lipoic acid alleviates colistin nephrotoxicity in rats. Hum. Exp. Toxicol..

[34] Liu Y., Zhang R., Velkov T., Shen J., Tang S., Dai C. (2025). Corynoxeine supplementation ameliorates colistin-induced kidney oxidative stress and inflammation in mice. Antioxidants.

[35] Kimura H., Kamiyama K., Imamoto T., Takeda I., Masunaga S., Kobayashi M., Mikami D., Takahashi N., Kasuno K., Sugaya T. (2022). Fenofibrate reduces cisplatin-induced apoptosis by inhibiting the p53/Puma/Caspase-9 pathway and the MAPK/Caspase-8 pathway rather than by promoting autophagy in murine renal proximal tubular cells. Biochem. Biophys. Rep..

[36] Jiang M., Wei Q., Wang J., Du Q., Yu J., Zhang L., Dong Z. (2006). Regulation of PUMA-α by p53 in cisplatin-induced renal cell apoptosis. Oncogene.

[37] Ordooei Javan A., Shokouhi S., Sahraei Z. (2015). A review on colistin nephrotoxicity. Eur. J. Clin. Pharmacol..

[38] Naraki K., Ghasemzadeh Rahbardar M., Razavi B.M., Aminifar T., Khajavi Rad A., Amoueian S., Hosseinzadeh H. (2024). The power of trans-sodium crocetinate: Exploring its renoprotective effects in a rat model of colistin-induced nephrotoxicity. Naunyn-Schmiedeberg’s Arch. Pharmacol..

[39] Jeong B.Y., Park S.-R., Cho S., Yu S.-L., Lee H.Y., Park C.G., Kang J., Jung D.-Y., Park M.H., Hwang W.-M. (2018). TGF-β-mediated NADPH oxidase 4-dependent oxidative stress promotes colistin-induced acute kidney injury. J. Antimicrob. Chemother..

[40] Li M. (2021). The role of P53 up-regulated modulator of apoptosis (PUMA) in ovarian development, cardiovascular and neurodegenerative diseases. Apoptosis.

[41] Samodelov S.L., Visentin M., Gai Z., Häusler S., Kullak-Ublick G.A. (2019). Renal glycosuria as a novel early sign of colistin-induced kidney damage in mice. Antimicrob. Agents Chemother..

[42] (2011). National Research Council Committee for the Update of the Guide for the, C.; Use of Laboratory, A. The National Academies Collection: Reports funded by National Institutes of Health. Guide for the Care and Use of Laboratory Animals.

[43] Yousef J.M., Chen G., Hill P.A., Nation R.L., Li J. (2011). Melatonin attenuates colistin-induced nephrotoxicity in rats. Antimicrob. Agents Chemother..

[44] Uchiyama T., Delude R.L., Fink M.P. (2003). Dose-dependent effects of ethyl pyruvate in mice subjected to mesenteric ischemia and reperfusion. Intensive Care Med..

[45] Baraka N.A.-F., Ahmed N.F., Hussein S.I. (2022). The effect of Rutin hydrate on Glucocorticoids induced osteoporosis in mandibular alveolar bone in Albino rats (Radiological, histological and histochemical study). Saudi Dent. J..

[46] Bozkus F., San I., Ulas T., Iynen I., Yesilova Y., Guler Y., Aksoy N. (2013). Evaluation of total oxidative stress parameters in patients with nasal polyps. Acta Otorhinolaryngol. Ital..

[47] Arifin W.N., Zahiruddin W.M. (2017). Sample size calculation in animal studies using resource equation approach. Malays. J. Med. Sci..

